# Interoceptive Awareness Is Negatively Related to the Exteroceptive Manipulation of Bodily Self-Location

**DOI:** 10.3389/fpsyg.2020.562016

**Published:** 2020-12-04

**Authors:** Robin Bekrater-Bodmann, Ruben T. Azevedo, Vivien Ainley, Manos Tsakiris

**Affiliations:** ^1^Department of Cognitive and Clinical Neuroscience, Central Institute of Mental Health, Medical Faculty Mannheim, Heidelberg University, Mannheim, Germany; ^2^Department of Psychology, Royal Holloway, University of London, Egham, United Kingdom; ^3^Department of Psychology, University of Kent, Canterbury, United Kingdom; ^4^Warburg Institute, School of Advanced Study, University of London, Bloomsbury, United Kingdom; ^5^Department of Behavioural and Cognitive Sciences, Faculty of Humanities, Education and Social Sciences, University of Luxembourg, Luxembourg

**Keywords:** dissociation, heartbeat, illusion, interoception, multimodal stimulation, out-of-body experience

## Abstract

The perception of being located within one’s body (i.e., bodily self-location) is an essential feature of everyday self-experience. However, by manipulating exteroceptive input, healthy participants can easily be induced to perceive themselves as being spatially dislocated from their physical bodies. It has previously been suggested that interoception, i.e., the processing of inner physiological signals, contributes to the stability of body representations; however, this relationship has not previously been tested for different dimensions of interoception and bodily self-location. In the present study, using an advanced automatized setup, we systematically manipulated participants’ perspective of their own body (first- vs third-person perspective) as well as the synchrony of visuotactile stimulation (synchronous vs asynchronous). The malleability of bodily self-location was assessed using a questionnaire targeting in-body and out-of-body experiences. Participants also performed a heartbeat discrimination task to assess their interoceptive accuracy (behavioral performance), interoceptive sensibility (confidence in their interoceptive abilities), and interoceptive awareness (meta-cognitive representation of interoceptive signals). Bodily self-location was significantly influenced by perspective, with third-person perspective being associated with stronger out-of-body experiences compared to first-person perspective. Furthermore, there was a significant perspective × stimulation interaction, with subsequent analyses showing that participants reported out-of-body experiences particularly under third-person perspective combined with synchronous visuotactile stimulation. Correlation and regression analyses revealed that meta-cognitive interoceptive awareness was specifically and negatively related to the exteroceptively mediated malleability of body experiences. These results indicate that the perception of the self being located within one’s body relies on the interaction of exteroceptive input and higher-order interoceptive abilities. This has implications for theoretical considerations about the bodily self in health as well as for the understanding of disturbed bodily self-processing in clinical contexts.

## Introduction

The perception of being located within one’s body, i.e., bodily self-location, is an essential feature of bodily self-consciousness (Blanke and Metzinger, 2009), describing the perception of oneself as an embodied agent with a first-person perspective. The processes that underlie bodily self-location are anything but trivial. At any given time, a variety of sensory signals have to be processed simultaneously and integrated into a corresponding percept, resulting in the experience of the self being located within the borders of the body. These processes appear to be abnormal under certain clinical and non-clinical conditions. For example, mental pathologies such as dissociative disorders, post-traumatic stress disorder, or borderline personality disorder are accompanied by strong dissociative experiences (Lyssenko et al., 2018), which can involve aberrant bodily self-location (e.g., Stiglmayr et al., 2003). Interestingly, 5% of the general population also report having such experiences at least once in their lifetimes (Ohayon, 2000), indicating that this unusual mode of locating the self in respect to the body is somehow part of the common repertoire of human perception.

Remarkably, the perceptual processes underlying bodily self-location can be easily manipulated by the application of unusual multimodal exteroceptive input (Ehrsson, 2007). In this kind of experiment, the participant wears a head-mounted display transmitting a streaming video signal recorded by a camera placed behind the subject. Thus, this setup creates the visual impression that the subject is directly watching his or her own back, from a third-person’s perspective. If the subject’s real chest and the space under the camera (i.e., the “chest” of the illusory body) are now touched synchronously in a sweeping motion, participants report the sensation of being spatially dislocated from their own body (Ehrsson, 2007). Since this unusual visuotactile condition is sufficient to elicit so-called out-of-body experiences, bodily self-location has been proposed to be fully mediated by exteroceptive input.

However, interoception, defined as the processing of inner physiological signals, represents another important source for an individual’s body representation. Interoception has been linked to a variety of psychological functions, such as emotion processing (e.g., Craig, 2004), social cognition (e.g., Ferri et al., 2013), or self-awareness (e.g., Ainley et al., 2012). In experimental setups, interoception is generally assessed by accuracy in identifying one’s own cardiac activity, be it by a task to mentally track one’s heartbeats (Schandry, 1981) or to discriminate whether a train of acoustic stimuli is synchronous or asynchronous with respect to one’s true heartbeats (Whitehead et al., 1977). Trait interoceptive accuracy, measured by individual performance in a heartbeat-tracking task, has been inversely linked to proneness to the rubber hand illusion (Tsakiris et al., 2011; see also Suzuki et al., 2013), which is a setup for the induction of illusory body-part ownership, by the application of synchronous visuotactile stimulation to one’s own hidden hand and a visible rubber hand (Botvinick and Cohen, 1998). This finding has been interpreted as indicating that the stability of one’s body representation is—at least partly—interoceptively mediated, such that good interoceptive abilities are associated with less proneness of body experience to be influenced by unusual exteroceptive input. Since the experimental induction of both the rubber hand illusion and out-of-body experiences appears to rely on similar capabilities for sensory integration (Olivé and Berthoz, 2012), a relationship between interoceptive abilities and more global bodily self-location is likely.

Recent studies have identified several distinct interoceptive dimensions (Garfinkel and Critchley, 2013), each characterized by different mental processes. While *interoceptive accuracy* refers to objective performance in tests of interoception, *interoceptive sensibility* describes a dispositional tendency for subjective beliefs about one’s own interoceptive abilities, and *interoceptive awareness* characterizes the metacognitive representation of one’s own interoceptive abilities, which can be assessed by applying signal detection theory using both interoceptive accuracy and sensibility measures. The validity of these measures has been recently demonstrated empirically (Garfinkel et al., 2015; Forkmann et al., 2016), indicating that they reflect relatively distinct dimensions of interoception. For example, there is evidence that the higher-order dimension of interoceptive awareness is a particular indicator of interoceptive abilities across organ-specific axes, such as cardiac and respiratory modalities (Garfinkel et al., 2016a). Thus, it is likely that relating these different measures of interoception to bodily self-location might reveal new insights into the underlying mechanisms.

In the present study, we implemented an advanced setup for the experimental manipulation of bodily self-location as well as a heartbeat discrimination task in a sample of healthy participants. In addition to the induction of out-of-body experiences by third-person perspective (3PP) that have been previously explored (Ehrsson, 2007), we introduced a novel condition in which participants were induced to perceive the scene from a first-person perspective (1PP), eliciting “normal” but still illusory in-body experiences that do not affect the location of the self in respect to the body. Our main hypotheses were that (a) synchronous visuotactile stimulation in 3PP compared to 1PP would induce distortions of bodily self-location and that (b) lower interoceptive abilities, in particular lower metacognitive interoceptive awareness, would be associated with more malleable bodily self-location.

## Materials and Methods

### Participants

There were 54 participants (42 females). Since it has been shown that accuracy in heartbeat discrimination is inversely linked to age (Khalsa et al., 2009), we checked our sample for extreme values (>3 times the interquartile range) and removed three subjects from the analysis. Body mass index (BMI) has also been found to be negatively related to interoceptive abilities (Herbert and Pollatos, 2014); however, taking into account five missing values for this measure (based on participants’ self-reports), no extreme values were observed. The final sample (*n* = 51; 40 females) had an age of *M* = 20.18 years (*SD* = 1.60) and a BMI of *M* = 22.94 (*SD* = 4.13). All except two participants declared themselves right-handed. No participant reported a history of psychiatric or neurological disorders, cardiovascular problems, chronic somatic or mental disorders, or chronic pain. All participants had normal or corrected-to-normal vision. Since the head-mounted display used in the present study (see below) was not compatible with conventional glasses, we only included contact lens users or participants whose uncorrected eyesight fell into the range for which the head-mounted display could adapt (i.e., −5 to +2 dpt). All participants gave written informed consent before participating. The study was approved by the ethics review board of Royal Holloway, London University, and adhered to the Helsinki Declaration of 1975, as revised in 2008.

### Setup for the Experiment Manipulating Bodily Self-Location

The setup for illusion induction was adapted from earlier work (Ehrsson, 2007). In our study, the participant was seated in a chair in the middle of a sparsely equipped room (about 3.5 × 4 m), wearing a head-mounted display (Cinemizer OLED 3D Multimedia Glasses, Carl Zeiss AG, Oberkochen, Germany). The head-mounted display received output from a video camera (Full HD Camcorder HC-X810, Panasonic Corporation, Kadoma, Japan), equipped with a 3D additional lens (VW-CLT2, Panasonic Corporation, Kadoma, Japan), which was mounted on a tripod at the eye level of the seated participant. The tripod was located 120 cm behind the participant so that—when in the active mode—the participant could see his or her own upper back, shoulders, and back of the head. The second tripod equipped with a stepper motor and brush (i.e., the visual brush) was placed in front of the camera, at a distance of 20 cm from the 3D lens. The level of the tripod was adjusted until one third of the brush’s bristles was in view of the camera. The whole setup was placed such that the walls in front of, and behind, the participants were at a distance of 120 cm (with reference to the participant’s chair and the video camera, respectively). We attached a fixation cross to the front wall at eye level. The first tripod (with the tactile brush) was placed in such a way that the brush was able to apply tactile stimulation to the participant’s upper chest. The angle and position of the tactile brush was individualized for each participant, and the use of soft bristle brushes minimized friction. In order to ensure a similar surface of stimulation across individuals, we applied a skin-compatible, adhesive, thin film (Suprasorb F, Lohmann & Rauscher, Rengsdorf, Austria) to the area of stimulation. Piloting showed that the tactile sensation was not affected by this film.

**FIGURE 1:**
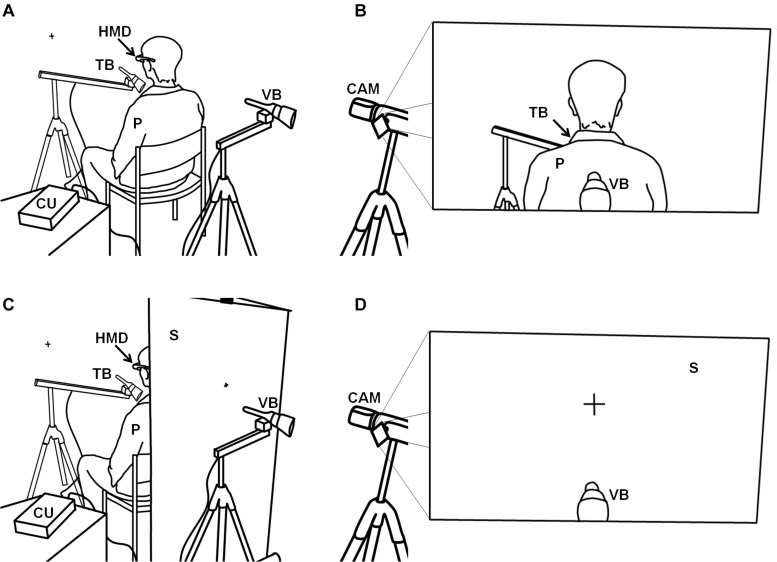
Illustration of the setup for the bodily self-location experiment. **(A)** The setup for the third-person perspective conditions with **(B)** the visual output received by the head-mounted display and perceived by the participant. **(C)** Setup for the first-person perspective conditions with **(D)** the visual output received by the head-mounted display and seen by the participant. P, participant; HMD, head-mounted display; TB, tactile brush; VB, visual brush; CAM, video camera; CU, control unit; S, screen.

In contrast to the original setup (Ehrsson, 2007) in which tactile stimulation was applied manually, tactile stimulation in the present study was applied using two stepper motors, each equipped with a soft brush, thus minimizing social interaction between the participant and the experimenter and maximizing standardization. Both stepper motors were attached to a swivel arm, each of which was in turn attached to a tripod. A control unit equipped with Arduino micro-controllers controlled both motors independently, enabling us to implement two conditions of the factor stimulation: (a) both stepper motors moved synchronously (sync condition), performing a back-and-forth movement by about 45°, or (b) both stepper motors moved asynchronously (async condition), i.e., such that as one brush moved, the other was inactive. In both modes, the brushes moved with a frequency of about 0.5 Hz. The setup is shown in Figure 1A, with the participant’s view illustrated in Figure 1B.

By using this setup, we were able to implement two conditions of the factor perspective, operationalizing either the condition 3PP (as used by Ehrsson, 2007, and described above) or 1PP, during which the setup differed in the single but important point that we placed a white screen between the participant’s chair and the visual brush, at a distance of 55 cm from the 3D objective and thus behind the participant (Figure 1C). In the two 1PP conditions (sync and async), we attached a fixation cross to the screen, whose size was adjusted to resemble the size of the fixation cross that is visible on the wall in the 3PP conditions. In other words, the visual scene in the two 1PP conditions was identical to that observable by the seated participants in the two 3PP conditions, before they put on the head-mounted display (Figure 1D). Thus, we implemented a full-factorial 2 (perspective, 1PP vs 3PP) × 2 (stimulation, sync vs async) experimental design, with the four conditions 1PP_sync_, 1PP_async_, 3PP_sync_, and 3PP_async_ presented in a randomized order.

### Procedure

The participants were briefly given an overview of the experimental procedure before they were asked to sign the consent form. They were then seated in the chair and equipped with the adhesive film and the head-mounted display. The participants were asked to sit in a relaxed and still position during the experiment. They were told to keep their eyes closed until the experimenter verbally requested them to open their eyes.

The sequence of events for each of the four stimulation blocks (i.e., the four experimental conditions, see above) was identical. First, the experimenter switched on the stepper motors and adjusted the brushes. Then he switched on the head-mounted display and asked the participant to open their eyes. During stimulation with the brushes, the participant was asked to observe the back of their head (in 3PP conditions) or the fixation cross at an identical position (in 1PP conditions; see Figure 1). After 90 s, the experimenter asked the participants to close their eyes, after which he switched off the stepper motors and removed the head-mounted display.

The participant then completed a questionnaire, starting with control items asking whether the participant had seen their body or a white wall during the last stimulation block and whether the stimulation applied had been synchronous or asynchronous. Participants were 100% correct in stating whether they had observed themselves or the “wall” (i.e., the screen). In all but four trials, participants correctly identified the stimulation as having been synchronous or asynchronous (i.e., 98.04% correct). Other items of the questionnaire were presented in randomized order and asked for “normal” (i.e., locating the self within the body) and aberrant (i.e., locating the self outside the body) body experiences during the trial, in terms of in-body and out-of-body experiences, respectively. Out-of-body experiences were assessed with two items, i.e., item #1 “I felt as if I was located outside my physical body” and item #2 “It felt as if I was sitting behind myself.” In-body experiences were assessed with two other items, i.e., item #3 “I felt as if my body belonged to me” and item #4 “I felt as if I was connected to my body.” Finally, in order to obtain a measure of the general vividness of the induced illusionary experiences, we included two further items, i.e., item #5 “I felt as if the scene I observed was directly in front of me” and item #6 “It seemed as if the touch I felt was caused by the brush I saw.” All items were designed to obtain valid responses in both the 3PP and the 1PP conditions and were tested in a pilot study (unpublished data). Responses were measured with a visual analog scale, ranging from 0 (*not at all*) to 100 (*very strong*). After the four randomly presented stimulation blocks, the interoceptive discrimination task was administered, as described below.

### Interoceptive Discrimination Task

Participants were equipped with three electrocardiography electrodes (ADInstruments PowerLab 8/35 and Bio Amp 132)^1^ placed in a modified lead II chest configuration: two electrodes were positioned underneath the left and right collarbones and another one on the participant’s lower back on the left side. The signal was recorded with a sampling rate of 1,000 Hz, and a hardware band-pass filter between 0.3 and 1,000 Hz was applied. Participants then performed the interoceptive discrimination task (Whitehead et al., 1977): on each trial, the subjects heard 10 sounds, each set of which was either synchronous with their own heartbeat (200 ms after the R-peak) or asynchronous (500 ms after the R-peak; Kleckner et al., 2015). There were 20 synchronous and 20 asynchronous trials, in randomized order. Subjects had to indicate on each trial whether the external sounds were synchronous or asynchronous with their own heartbeat. They also had to rate the confidence in their decision on each trial using a visual analog scale ranging from 0 (*not confident*) to 100 (*confident*). Participants were asked to sit relaxed with their hands on their thighs and their legs uncrossed. They could freely choose whether they preferred to do the task with their eyes open or closed. Due to artifacts (visual inspection), we removed a total of 21 trials (i.e., 1.03% of all trials).

### Data Analysis

#### Ratings of Body Experiences and Its Malleability by Exteroceptive Input (Exteroception Index)

##### Ratings of In-Body and Out-of-Body Experiences

Intensity of out-of-body experiences (i.e., locating the self outside the body) was calculated as the mean of questionnaire items #1 and #2, and intensity of in-body experiences (i.e., locating the self within the body) was defined as the mean of items #3 and #4. In-body and out-of-body experiences were separately analyzed with analyses of variance (ANOVAs) for repeated measures, with the two factors *perspective* (1PP vs 3PP) and *stimulation* (sync vs async). For all ANOVAs, we report test statistics, *p*-values, and effect size (partial η^2^). Whenever an interaction effect was significant, we further applied *post hoc* paired-sample *t*-tests for which we also report effect sizes (Cohen’s *d*). In these, as well as all other analyses reported below, the tests were performed two-tailed. The two types of body experiences were also checked for intercorrelation using Spearman correlations. These, and all the analyses described below, were performed with IBM SPSS v26.

##### Exteroception Index

For each condition, we subtracted the ratings of out-of-body experiences (the mean of items #1 and #2) from the ratings of in-body experiences (mean of items #3 and #4), such that positive values indicate net co-location of the body and the self and negative values indicate net dislocation of the body and the self (see Table 1). This procedure is based on a pilot study revealing a significant negative relationship between both measures (unpublished data), suggesting content validity. The resulting scores—representing bodily self-location for each condition on the continuum *self located outside the body* and *self located inside the body*—were used in the following formula, to create an *exteroception index* (potential range: −100 to +100), representing the extent to which bodily self-location is malleable by the manipulation of exteroceptive input:

$$\frac{1}{4}\,\left[\left(1\,{\text{PP}}_{\text{sync}}-1\,{\text{PP}}_{\text{async}}\right)-\left(3\,{\text{PP}}_{\text{sync}}-3\,{\text{PP}}_{\text{async}}\right)\right]$$

**TABLE 1:** Mean (*M*) values and standard deviations (*SD*) for in-body experiences, out-of-body experiences, and net body experiences, the net body experience, and the latter of which represent the in-body minus out-of-body experiences, per condition.

Condition	In-body experiences *M* (*SD*)	Out-of-body experiences *M* (*SD*)	Net body experience *M* (*SD*)
1PP_sync_	83.05 (15.63)	12.31 (13.63)	70.74 (26.30)
1PP_async_	77.64 (20.93)	16.36 (20.68)	61.27 (37.12)
3PP_sync_	59.85 (22.74)	65.20 (24.81)	−5.34 (42.64)
3PP_async_	59.76 (24.35)	56.12 (26.37)	3.65 (44.36)

*1PP, first-person perspective; 3PP, third-person perspective; sync, synchronous visuotactile stimulation; async, asynchronous visuotactile stimulation.*

The advantage of this index is that it uses all four of our conditions to represent a measure of the extent to which an individual is using both *perspective* and *stimulation* as sources of information with which to establish/maintain bodily self-location. The operative word here is “both,” since high values can only be achieved if both the type of perspective and the type of stimulation influence the location of the self in respect to the body. Hypothetically, an exteroception index of +100 would indicate maximum malleable bodily self-location by consistent exteroceptive input, in terms of strong in-body experiences when in 1PP_sync_ and strong out-of-body experiences when in 3PP_sync_. Asynchronous visuotactile input, on the other hand, would reverse this response. Such a hypothetical participant’s body experience would thus adjust completely to consistent exteroceptive input. By contrast, an exteroception index of 0 would indicate that exteroceptive input has no effects on bodily self-location or that this hypothetical participant’s body experience is relying solely on one exteroceptive source (either *perspective* or *stimulation*). An exteroception index of −100 would not only indicate that the participant’s bodily self-location does not comply at all with consistent exteroceptive input but that there is some kind of psychological resistance to conflicting exteroceptive input, resulting in strong co-location of the body and the self under 1PP_async_ and strong dislocation of the body and the self under 3PP_async_ conditions.

The exteroception index was tested against 0 using a one-sample *t*-test to check whether, on average, bodily self-location was experimentally manipulated by exteroceptive input.

#### Vividness of Body Experiences

Vividness of body experiences has generally been included in previous calculations of illusion scores in experiments on body perception (e.g., Botvinick and Cohen, 1998; Ehrsson, 2007). Calculating this as the mean of questionnaire items #5 and #6, we entered this measure in an ANOVA, similarly as for the body experience scores. The purpose was a manipulation check for whether the vividness of induced illusory experiences would differ as a result of visuotactile stimulation (i.e., with expected higher vividness in the sync compared to the async conditions), but not as a function of perspective alone (i.e., 1PP vs 3PP).

#### Interoceptive Measures

For analyzing the three different dimensions of interoception, i.e., interoceptive accuracy, interoceptive sensibility, and interoceptive awareness, we followed the procedure described by Garfinkel et al. (2015). First, we calculated the relative number of correct heartbeat discrimination trials, i.e., interoceptive accuracy, resulting in an individual value potentially ranging from 0 (*no correct responses*) to 1 (*all responses were correct*). Second, interoceptive sensibility was defined as mean confidence in one’s own interoceptive performance, which was divided by 100 in order to make this measure comparable to the others [i.e., 0 (*very unconfident*) to 1 (*very confident*)]. Third, for determining interoceptive awareness, we again performed analyses in accordance with Garfinkel et al. (2015): we applied receiver operating characteristic (ROC) curve analysis (Green and Swets, 1966), quantifying the extent to which confidence in interoception predicts interoceptive accuracy. The area under the ROC curve thus gives a measure for the association between confidence and performance, by plotting the hit rate (performance is correct *and* the participant is highly confident in his or her performance) against the false alarm rate (performance is *in*correct while the participant’s confidence is still high). Since this method accounts for the individual bias in reporting high or low confidence, this measure represents a valid indicator of metacognitive awareness for interoceptive processes (Garfinkel and Critchley, 2013; Garfinkel et al., 2015).

For each of these measures, we applied a one-sample *t*-test with the test value 0.5 to test the hypothesis that the group as a whole performed above chance (interoceptive accuracy and awareness) or had above 50% confidence in their interoceptive abilities (interoceptive sensibility), respectively.

#### Association Between Bodily Self-Location and Interoceptive Dimensions

Across the total sample, we correlated the exteroception index with the interoceptive measures by applying two-tailed Pearson correlations. Furthermore, we used multiple linear regression to better evaluate our results. Interoceptive measures, i.e., interoceptive accuracy, interoceptive sensibility, and interoceptive awareness, were entered (simultaneous entry). The ANOVA testing for significance of explained variance (*R*^2^) is reported, as well as the adjusted *R*^2^. For each regressor, the unstandardized coefficient *B* and its standard error *SE* are reported, along with the standardized regression coefficient β and the respective *p*-value.

Since out-of-body experiences were particularly affected by perspective and stimulation (see section “Body Experience Ratings and the Exteroception Index” below), the analyses described above were repeated for the effect between 3PP_sync_ and 3PP_async_ conditions.

## Results

### Body Experience Ratings and the Exteroception Index

Descriptive statistics for the in-body and out-of-body experiences are provided in Table 1 (mean values for each item are further shown in Supplementary Figures 1A,B in the Supplementary Material). We performed a 2 × 2 ANOVA for repeated measurements, entering the two factors *perspective* (1PP vs 3PP) and *stimulation* (sync vs async). For in-body experiences, we found a significant main effect of the factor *perspective* (*F*_1_,_50_ = 38.95, *p* < 0.001, and η^2^ = 0.44), but not for the factor *stimulation* (*F*_1_,_50_ = 1.35, *p* = 0.25, and η^2^ = 0.03). There was no interaction (*F*_1_,_50_ = 2.39, *p* = 0.13, and η^2^ = 0.05). For out-of-body experiences, there was a significant main effect for the factor *perspective* (*F*_1_,_50_ = 172.41, *p* < 0.001, and η^2^ = 0.78), but not for *stimulation* (*F*_1_,_50_ = 1.17, *p* = 0.28, and η^2^ = 0.02). However, there was a significant interaction (*F*_1_,_50_ = 9.02, *p* = 0.004, and η^2^ = 0.15), which was caused by significantly stronger out-of-body experiences in the 3PP_sync_ condition compared to the 3PP_async_ condition (*t*_50_ = 2.53, *p* = 0.01, and *d* = 0.35); there were no significant differences in the 1PP_*sync*_ condition compared to the 1PP_*async*_ condition (*t*_50_ = −1.48, *p* = 0.14, and *d* = 0.23).

There were highly significant, negative correlations between in-body and out-of-body experiences (*r*_49_ between −0.53 and −0.64, all *p* < 0.001 for each condition; *r*_49_ = −0.63, *p* < 0.001 across conditions). This indicates that the ratings for in-body experiences represent at least partly the opposite to the ratings for out-of-body experiences and *vice versa*, suggesting valid assessments.

**FIGURE 2:**
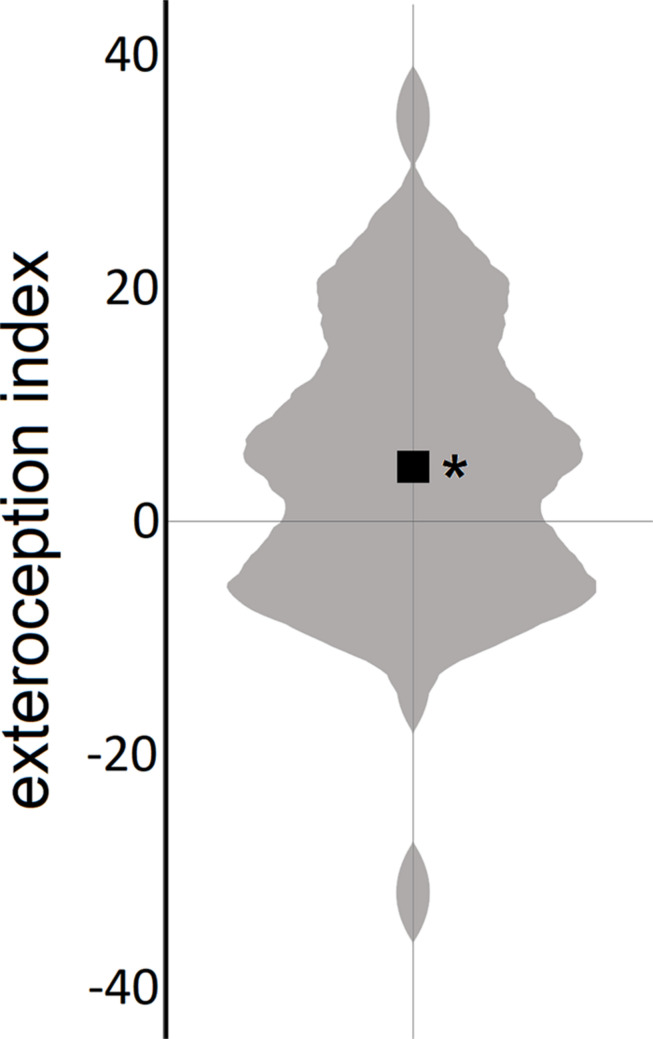
Experimental manipulation of body self-location. Violin plot of the exteroception index (the black square represents the mean); positive values indicate malleable bodily self-location by exteroceptive input. *p < 0.05.

The mean value of the exteroception index (*M* = 4.61, *SD* = 12.21) differed significantly from 0 (*t*_50_ = 2.70, *p* = 0.01, and *d* = 0.38), indicating that, on average, bodily self-location was successfully manipulated by exteroceptive input in the experiment (Figure 2).

### Vividness of Body Experiences

**FIGURE 3:**
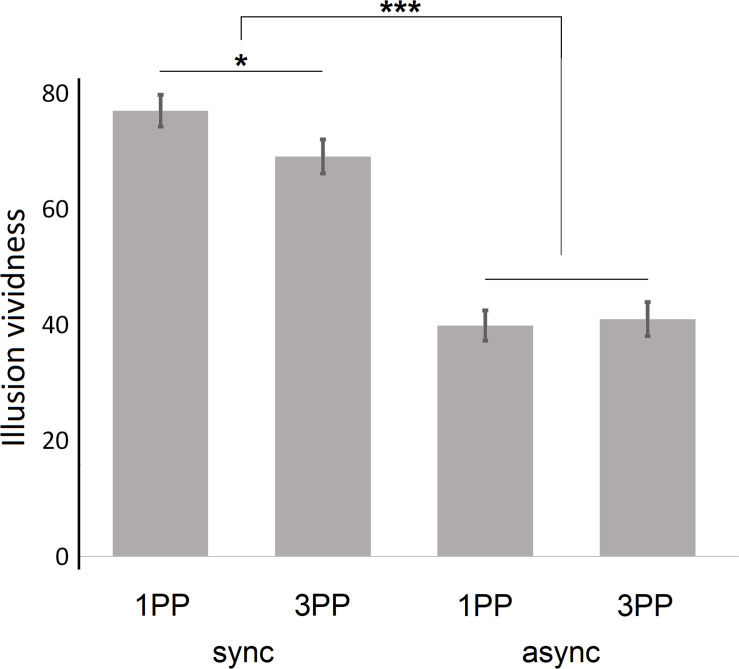
General vividness of illusory sensations; given are the means; error bars indicate the standard error of the mean. 1PP, first-person perspective; 3PP, third-person perspective; sync, synchronous visuotactile stimulation; async, asynchronous visuotactile stimulation; *p < 0.05; ***p < 0.001.

Mean vividness was higher for synchronous conditions (*M* = 76.91, *SD* = 19.87 for 1PP_sync_; *M* = 69.02, *SD* = 20.96 for 3PP_sync_) than for asynchronous conditions (*M* = 39.81, *SD* = 18.86 for 1PP_async_; *M* = 40.93, *SD* = 21.11 for 3PP_async_; see Figure 3).

We performed a 2 × 2 ANOVA for repeated measurements, again entering the two factors *perspective* and *stimulation*. We found a significant main effect on vividness of the factor *stimulation* (*F*_1_,_50_ = 146.42, *p* < 0.001, and η^2^ = 0.75), but no main effect of the factor *perspective* (*F*_1_,_50_ = 1.80, *p* = 0.19, and η^2^ = 0.03). There was a significant *perspective* × *stimulation* interaction (*F*_1_,_50_ = 5.91, *p* = 0.02, and η^2^ = 0.11); subsequent *post hoc* paired-samples *t*-tests revealed that the interaction was caused by a significant difference between 1PP_sync_ and 3PP_sync_ (*t*_50_ = −2.41, *p* = 0.02, and *d* = 0.39), which was not present when comparing 1PP_async_ and 3PP_async_ (*t*_50_ = 0.38, *p* = 0.71, and *d* = 0.06).

Mean values for individual vividness items per condition are shown in Supplementary Figure 1C in the Supplementary Material.

### Dimensions of Interoception

Regarding interoceptive accuracy, the total group performed above chance in correctly distinguishing between synchronous and asynchronous trains of sounds and their own heartbeats (*M* = 0.55, *SD* = 0.13; *t*_50_ = 2.89, *p* = 0.006, and *d* = 0.38). Confidence in their own interoceptive abilities (i.e., interoceptive sensibility) was significantly above 0.5 (*M* = 0.61, *SD* = 0.12; *t*_50_ = 6.83, *p* < 0.001, and *d* = 0.92). Metacognitive interoceptive awareness, measured using ROC curve analysis of accuracy and confidence in heartbeat discrimination task data, reached above-chance significance across the whole group (*M* = 0.56, *SD* = 0.10; *t*_50_ = 4.25, *p* < 0.001, and *d* = 0.60).

### Relationship Between Interoceptive Dimensions and the Exteroception Index

**FIGURE 4:**
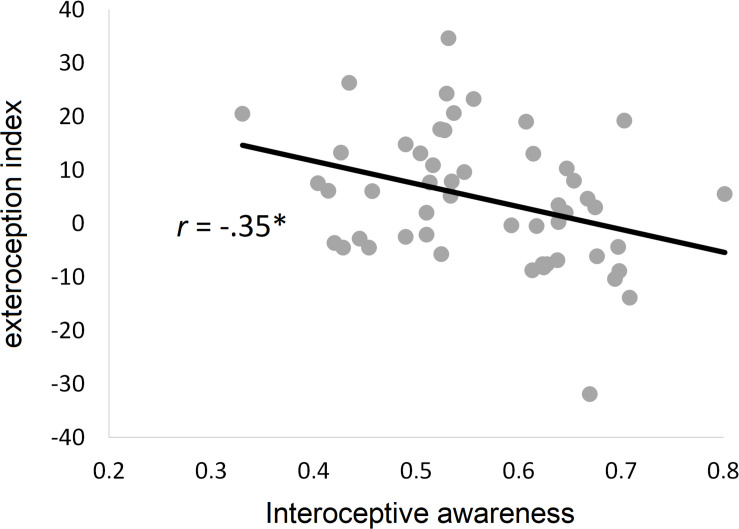
Relationship between interoceptive dimensions and bodily self-location. Scatter plot and regression line for the relationship between malleability of bodily self-location by exteroceptive input (exteroception index) and interoceptive awareness (*r* = Pearson correlation coefficient). ^∗^*p* < 0.05.

While the exteroception index was not significantly correlated with interoceptive accuracy (*r*_49_ = −0.04, *p* = 0.77) or interoceptive sensibility (*r*_49_ = −0.22, *p* = 0.11), there was a significantly negative correlation with interoceptive awareness (*r*_49_ = −0.35, *p* = 0.01), indicating that the higher the interoceptive awareness, the less a participant’s bodily self-location is malleable by exteroceptive input. This relationship is shown in Figure 4.

The ANOVA for the regression analysis including all three interoceptive measures was significant, *F*_3_,_47_ = 3.56, *p* = 0.02, with an adjusted *R*^2^ of 13.3%. Only interoceptive awareness showed a significant association with the exteroception index (for statistical details, see Table 2).

**TABLE 2:** Results of regression analyses.

Criterion	Regressors	*B*	*SE*	β	*P*	*R*^2^	adjusted *R*^2^
Exteroceptive index	(constant)	35.82	11.70		0.004	0.19	0.13
	Interoceptive accuracy	20.17	14.83	0.21	0.18		
	Interoceptive sensitivity	–22.58	14.04	–0.22	0.11		
	Interoceptive awareness	–50.92	18.30	–0.41	0.008		
Out-of-body experiences (3PP_snyc_ minus 3PP_async_)	(constant)	74.67	24.60		0.004	0.18	0.13
	Interoceptive accuracy	44.21	31.19	0.22	0.16		
	Interoceptive sensitivity	–56.83	29.53	–0.26	0.06		
	Interoceptive awareness	–98.50	38.48	–0.38	0.01		

*3PP, third-person perspective; sync, synchronous visuotactile stimulation; async, asynchronous visuotactile stimulation; *R*^2^, explained variance; *B*, unstandardized coefficient; *SE*, standard error; β, standardized regression coefficient.*

Since the analysis of body experience ratings (see section “Body Experience Ratings and the Exteroception Index” above) revealed a significant *perspective* × *stimulation* interaction only for out-of-body experiences, the validity of the exteroception index has been challenged during the revision process. Thus, we repeated the correlation and regression analyses for the score representing this significant interaction effect (i.e., subtracting the out-of-body experience ratings from the 3PP_async_ condition from those obtained in the 3PP_sync_ condition; see Table 1). The pattern of correlations remained stable (*r*_49_ = −0.03, *p* = 0.83 for interoceptive accuracy; *r*_49_ = −0.26, *p* = 0.07 for interoceptive sensibility; *r*_49_ = −0.32, *p* = 0.02 for interoceptive awareness), and also the regression analysis results were comparable (*F*_3_,_47_ = 3.53, *p* = 0.02; adjusted *R*^2^ = 13.2%; for details, see Table 2), particularly regarding the specific association with interoceptive awareness, suggesting that exteroceptive manipulation predominantly affected perceived dislocation, rather than co-location, of the body and the self, which itself is specifically negatively associated with interoceptive awareness.

## Discussion

Bodily self-consciousness is composed of three key components, perceptually reflected by the sensation of having a body that (a) belongs to one’s self (self-identification), (b) is the locus from where an individual is experiencing the world (first-person perspective), and (c) is experienced as occupying a specific location in space (self-location; Blanke, 2012). These dimensions of bodily self-consciousness are tightly interlinked (Huang et al., 2017) and can be mediated by exteroception and/or interoception (e.g., Aspell et al., 2012, 2013). The experience of one’s self being located within one’s body, as assessed in the present study, represents the “normal” perceptual consequence of these processes. Both exteroceptive (Ehrsson, 2007; Lenggenhager et al., 2007) and interoceptive (Adler et al., 2014) processes have been identified as specifically contributing to the co-location of the body and the self.

In the present study, we further investigated the interrelationship between exteroception and interoception underlying the feeling that the self is located within the borders of one’s own body. Healthy subjects participated in an experiment composed of four conditions that systematically manipulated exteroceptive multimodal input in terms of *perspective* (1PP vs 3PP) and visuotactile *stimulation* (sync vs async), using an advanced setup based on the work of Ehrsson (2007). Participants were asked to complete a questionnaire that measured their level of perceived co-location (i.e., in-body experiences) or dislocation of their body and their self (i.e., out-of-body experiences). We found that *perspective* had a significant main effect on bodily self-location, with 1PP being associated with higher levels of co-location of the body and the self, while 3PP being associated with perceived dislocation. Particularly, for the unusual experience to be separated from one’s physical body, inconsistent multimodal exteroceptive input might play a crucial role: the out-of-body-specific significant *perspective* × *stimulation* interaction suggests that 3PP_snyc_ induced significantly stronger feelings of dislocation of the body and the self than 3PP_async_, indicating that visuotactile stimulation is particularly capable of modulating bodily self-location under unusual perspectival conditions.

We also calculated an “exteroception index” that reflects the degree to which a participant’s individual body experience adjusted to exteroceptive input. Correlating this index with our three dimensions of interoceptive abilities, assessed with a heartbeat discrimination task, showed that interoceptive awareness, i.e., the metacognitive representation of interoceptive abilities, was significantly negatively related to the exteroception index. This relationship was specific to interoceptive awareness, since interoceptive accuracy (in terms of behavioral accuracy in performance) and interoceptive sensibility (in terms of being confident in one’s own interoceptive abilities) did not show any similar correlation. Regression analyses further emphasized that interoceptive awareness was specifically associated with the malleability of bodily self-location by exteroceptive input. This indicates that better metacognitive interoceptive awareness is accompanied by a body percept that is less prone to being malleable by exteroceptive input. In other words, such participants’ perceptual systems rely more on higher-order interoceptive processes rather than on exteroception, resulting in a more stable body representation. The results also remained significant if we focused on the significant *perspective* × *stimulation* effect on out-of-body experiences, suggesting that dislocation, rather than co-location, is particularly relying on metacognitive interoceptive capabilities. These results are of importance for theoretical conceptions about bodily self-consciousness, as well as for the understanding of psychopathologies characterized by aberrant connections between the body and the self.

Our novel setup induced significantly higher levels of perceived co-location between the body and the self in 1PP conditions compared to 3PP conditions, emphasizing its exteroceptive basis. Braithwaite et al. (2017) recently showed that participants who report that they have out-of-body experiences, i.e., an extreme form of aberrant bodily self-location in their normal life, also display (not necessarily pathological) aberrations in the integration of exteroceptive sensory input. This is supported by our results. In our study, visuotactile stimulation interacted with perspective (1PP vs 3PP), albeit the effect sizes were rather small compared to other studies (e.g., Ehrsson, 2007). This might result from our exclusion of the vividness component—which has previously been included as an illusion marker in other studies on body experience (e.g., Botvinick and Cohen, 1998; Ehrsson, 2007)—and could have reduced the apparent effect of stimulation in the present study, given that vividness has been shown to be particularly associated with synchronous visuotactile stimulation. Furthermore, the small to absent effects of the factor *stimulation* on in-body experiences could reflect that this particular feature of everyday bodily self-consciousness is binary rather than continuous (cf. De Vignemont, 2011): if someone feels already being in his or her body, this perception cannot be easily increased by exteroceptive stimulation. Prospective studies have to further explore the dimensionality of in-body and out-of-body experiences.

This study adds to the existing empirical evidence that links the malleability of bodily self-consciousness to interoceptive abilities (e.g., Tsakiris et al., 2011; Adler et al., 2014). While the integration of sensory information across exteroceptive and interoceptive domains has previously been shown to modulate bodily self-consciousness (Suzuki et al., 2013), this has mainly been probed by using the rubber hand illusion paradigm. However, the rubber hand illusion and its derivatives (for a review, see Riemer et al., 2019) transfer the sense of self to an artificial body-part, while in the present study, we manipulated the perceived location of the self in respect to one’s *own* body, which might represent a distinct phenomenon relying on different neurocognitive mechanisms [see the discussion linked to the articles by Ehrsson (2007) and Lenggenhager et al. (2007)]. A further difference here to the rubber hand illusion might be that general bodily self-location represents a rather implicit level of bodily awareness. One previous study asked participants to localize their self in their body (Alsmith and Longo, 2014) and found participants pointing predominantly to the core of the body (the torso or the head) and not to the body’s periphery. Thus, there is reason to assume that co-location of the body and the self might be a more fundamental aspect of bodily self-consciousness than body-part ownership as tested by the rubber hand illusion.

Most importantly, our results indicate that higher-order, metacognitive interoceptive awareness, rather than behavioral performance on interoceptive accuracy or interoceptive sensibility, predicts the malleability of the bodily self-location. Interoceptive awareness, compared to the other interoceptive dimensions, seems to be a particularly reliable indicator of one’s own bodily states, since previous findings revealed that interoceptive awareness—in contrast to interoceptive accuracy—represents a relatively stable trait across physiological modalities (Garfinkel et al., 2016a). Thus, this particular interoceptive dimension might also play a role in the stability (or malleability) of body representations: the more reliable the information is from my body and the more I am aware of this reliability, the less dependent my perceptual system is on exteroceptive information. Accordingly, high cardiac interoceptive awareness could be a protective factor against aberrant bodily experiences. Whether other forms of interoceptive awareness (e.g., for respiratory signals; Garfinkel et al., 2016a) will show the same relationship remains open. However, one has to keep in mind that the amount of explained variance in the current regression and correlation analyses is rather small, which might emphasize the multifactorial origin of bodily self-consciousness. Prospective studies have to further elucidate the complex interplay of perceptual and cognitive factors.

Previous studies have investigated the neurophysiological mechanisms underlying the importance of interoception for body representations. Park et al. (2016) found that neural responses to heartbeats covary with changes in self-location. In accordance with those results, lesions of the right insula, which represent a key area for interoceptive processing (Critchley et al., 2004), decrease heartbeat awareness and alter self-location (Ronchi et al., 2015). In addition to the insula, higher-order brain areas such as the parietal cortex or the Rolandic operculum seem to integrate both interoceptive and exteroceptive signals in order to maintain bodily self-consciousness (Blefari et al., 2017; Park and Blanke, 2019; Salvato et al., 2020). Other results highlight the relevance of interoceptive information for the visual processing of the body (Ronchi et al., 2017), and this processes’ impact on brain areas involved in processing cognitive aspects of body representation. If replicated, the present results might further point to the frontal cortex: as Molenberghs et al. (2016) reported, the anterior medial prefrontal cortex seems to be specifically involved in metacognitive awareness, while this region has also been associated with switching between 1PP and 3PP taking (Ruby and Decety, 2003). Whether or not this region also mediates a trait-like coupling between exteroceptive and interoceptive sensory information by means of a malleable sense of bodily self-location, however, remains open.

Although we only included participants who did not report a history of psychopathology, our results might have implications for disorders associated with abnormalities in interoception and bodily self-consciousness. There is growing evidence that disturbed interoception may be important for dysfunctions in several conditions of disordered mental health (Khalsa et al., 2018). For example, borderline personality disorder patients, who frequently report dissociative body perceptions (Löffler et al., 2020), have been characterized by both abnormal integration of multimodal sensory input (Bekrater-Bodmann et al., 2016) and disturbed neural representation of interoceptive signals (Müller et al., 2015). Accordingly, interoceptive abilities, particularly interoceptive awareness, have been proposed to play an etiological role in the development of this disorder (Löffler et al., 2018), probably via repeated invalidation experiences in early life. Further, recent studies suggest that discrepancies between interoceptive processing and beliefs about, or interpretation of, interoceptive signals might reflect body awareness and affective deficits in individuals with autism spectrum disorder (Garfinkel et al., 2016b; Shah et al., 2016). In this group, aberrant interoception has also been linked to proneness to the rubber hand illusion (Schauder et al., 2015), suggesting that an impaired interplay between exteroception, interoception, and the malleability of the body percept plays a role for certain psychopathologies characterized by disturbed self-representation. We propose that disturbed integration of interoceptive and exteroceptive inputs is potentially linked to higher cognitive functions, which may be the basis of abnormal (bodily) self-processing. Thus, therapeutic treatments for these disorders could consider techniques, such as mindfulness-based interventions, that focus not only on the ability to correctly attend to body signals but also on the change of metacognitive representations and related cognitions (Khalsa et al., 2018). Prospective studies could investigate whether interoceptive awareness might be trained in such settings and whether improvement would be accompanied by reductions in the frequency or intensity of aberrant body experiences.

Limitations of the present study include that in-body and out-of-body experiences exclusively rely on self-reports. It is possible that the setup itself induced response biases in the participants, which might reduce the validity of results. Previous studies have used behavioral (e.g., Lenggenhager et al., 2007), peripheral physiological (e.g., Ehrsson, 2007), or central neurophysiological measures (e.g., Ionta et al., 2011) as correlates of uncommon body experiences, which should also be included in future studies. Since we initially assumed that 1PP_sync_ mimics the “normal” experience of bodily self-location, we omitted to operationalize a further control condition in which participants’ body experiences would be assessed in the absence of any tactile stimulation (be it in 1PP or 3PP). Thus, it remains an open question how similar the experimentally manipulated processes, and the perceptions they give rise to, are to real-life embodied experiences. This missing external validation is of particular relevance, since the nature of the experiment (i.e., seeing a body in 3PP conditions, while seeing no body in 1PP conditions) could itself have affected the results. The fact that perspective, rather than stimulation, influenced the findings of in-body experience in the present study might point to such a confounding effect. Future studies should explore the validity of the present findings by including behavioral or physiological measures, such as skin temperature (Salomon et al., 2013), reaction times (Adler et al., 2014), or behavioral measures of sensory interference (Maselli and Slater, 2014).

### Conclusion

Our results suggest that exteroceptive input and higher-order interoceptive abilities both contribute to the stability or malleability of perceived co-location of the body and the self. The implications of these findings are of relevance for theoretical considerations as well as for treatment of disorders characterized by abnormal self-processing. The neurobiological underpinnings should be investigated in future studies.

## Data Availability Statement

The dataset analyzed for the current study is available from the corresponding author on reasonable request.

## Ethics Statement

The studies involving human participants were reviewed and approved by Ethics review board of Royal Holloway, London University. The patients/participants provided their written informed consent to participate in this study.

## Author Contributions

RB-B and MT conceived and planned the experiments. RB-B and RA developed the technical setup. RB-B carried out the experiments and performed the data analysis. RA, VA, and MT contributed to the interpretation of the results. RB-B wrote the first draft of the manuscript, and RA, VA, and MT provided critical feedback. All authors contributed to the article and approved the submitted version.

## Conflict of Interest

The authors declare that the research was conducted in the absence of any commercial or financial relationships that could be construed as a potential conflict of interest.

## Footnotes

1 http://www.adinstruments.com/
