# Supplementary material for: Interoceptive Awareness Is Negatively Related to the Exteroceptive Manipulation of Bodily Self-Location
Source: Front Psychol. 2020 Dec 4;11:562016. doi: 10.3389/fpsyg.2020.562016 (PMC7746809; doi:10.3389/fpsyg.2020.562016)
Supplement: Supplementary file 1 [file Image_1.pdf]

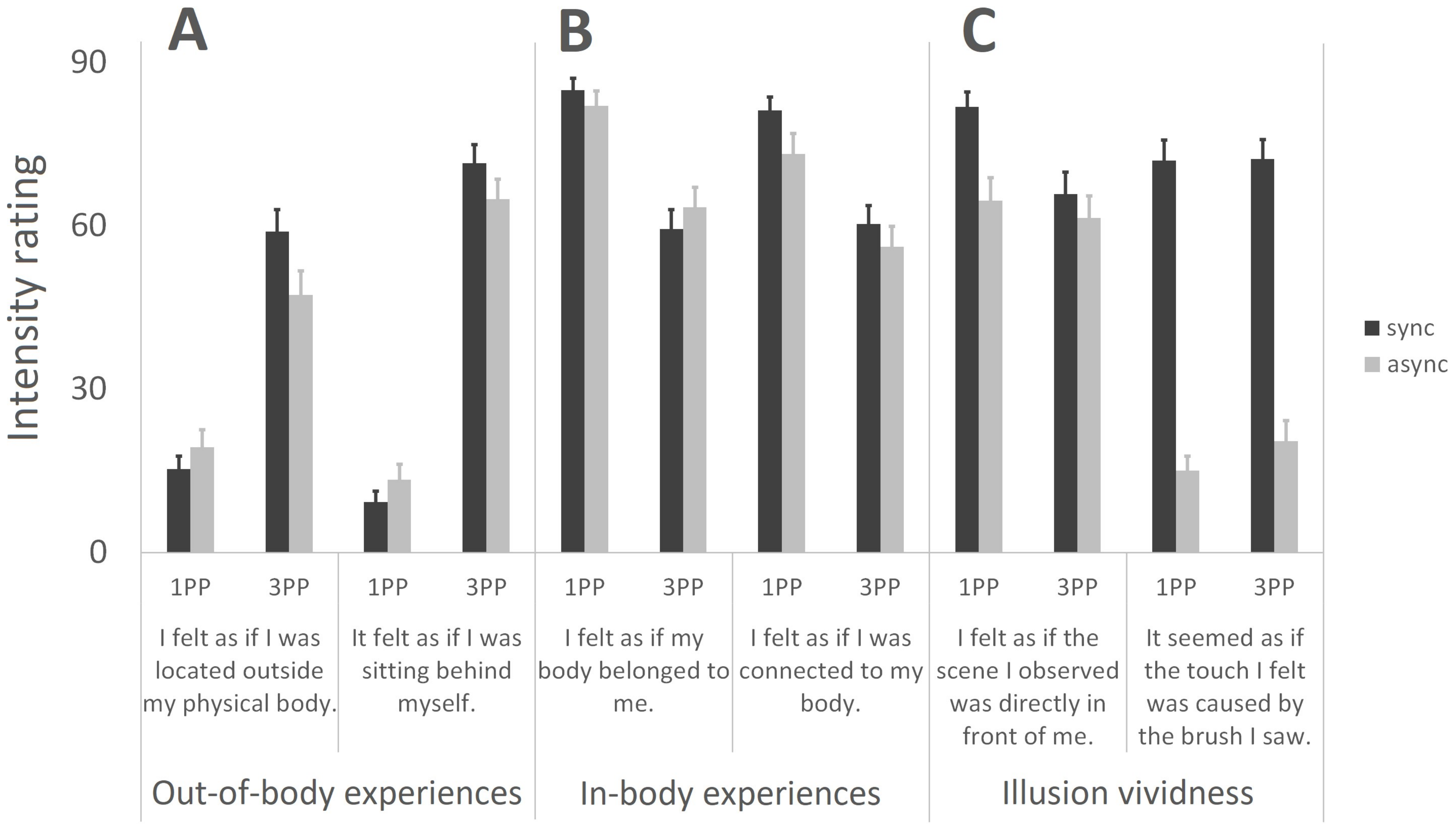

Figure S1: Ratings for individual items (#1-6). (A) out-of-body experiences; (B) in-body experiences; (C) general vividness of illusory experiences; given are the means, error bars indicate the standard error of the mean; 1PP = first-person perspective; 3PP = third-person perspective; sync = synchronous visuotactile stimulation; async = asynchronous visuotactile stimulation
